# Abortion and various associated risk factors in dairy cow and sheep in Ili, China

**DOI:** 10.1371/journal.pone.0232568

**Published:** 2020-10-30

**Authors:** Huan Zhang, Xiaoyu Deng, Buyun Cui, Zhiran Shao, Xiaoli Zhao, Qin Yang, Shengnan Song, Zhen Wang, Yong Wang, Yuanzhi Wang, Zhengfei Liu, Jinliang Sheng, Chuangfu Chen

**Affiliations:** 1 School of Animal Science and Technology, Shihezi University, Shihezi City, Xinjiang, China; 2 State Key Laboratory for Infectious Disease Prevention and Control, Collaborative Innovation Center for Diagnosis and Treatment of Infectious Diseases, National Institute for Communicable Disease Control and Prevention, Chinese Center for Disease Prevention and Control, Beijing, China; 3 School of Medicine, Shihezi University, Shihezi City, Xinjiang, China; 4 State Key Laboratory of Agricultural Microbiology, College of Veterinary Medicine, Huazhong Agricultural University, Wuhan City, China; University of Lincoln, UNITED KINGDOM

## Abstract

We studied livestock abortion and various associated risk factors in the Ili region of northwest China. Livestock abortion prevalence was estimated and correlated with infections (Brucellosis, Salmonellosis, *Mycoplasma* and *Chlamydia* seropositivity) and management (farming type and contact with other herds/flocks) risk factors. A total of 2996 serum samples (1406 cow, 1590 sheep) were identified by RBPT (Rose Bengal Plate Test) and c-ELISA (competitive-enzyme linked immunosorbent assay), and they showed the overall seroprevalence of brucellosis in the study area was cow 6.76%, sheep 9.50%. The seroprevalence of brucellosis in X county was cow 7.06%, sheep 9.12%; in H county was cow 11.70%, sheep 10.80%; and in Q county was cow 4.22%, sheep 9.11%. The overall seroprevalence of *Mycoplasma* in the study area was cow 3.20%, sheep 6.42%. The seroprevalence of *Mycoplasma* in X county was cow 3.39%, sheep 7.98%; in H county was cow 5.26%, sheep 9.97%; and in Q county was cow 2.11%, sheep 4.33%. The Odds ratio of brucellosis for cow and sheep, respectively, were 45.909 [95% CI 26.912–78.317, *P*<0.001] and 70.507 [95% CI 43.783–113.544, *P*<0.001] times higher than other abortion-related factors including mixed farming, contact with other flocks and *Mycoplasma* infection. A total of 54 samples, including aborted cow (22), sheep (30) fetuses and milk samples (2), were identified as *Brucella melitensis* (*B*. *melitensis*) positive. A total of 38 *Brucella* were isolated from 16 aborted cow, 20 sheep fetuses and 2 milk samples. All of these isolates were identified, and confirmed, as *B*. *melitensis*. A phylogenetic tree showed that the *Brucella* isolates closely matched the *B*. *melitensis* biovar 3 isolated in Inner Mongolia, China, and *B*. *melitensis* isolated from Norway and India. These results suggest that *B*. *melitensis* biovar 3 is the main pathogen responsible for cow and sheep abortion and also pose a human health risk. Additionally, livestock reproduction can also be influenced by *Mycoplasma* infection and managerial factors (farming type and contact with other herds/flocks), especially in remote areas.

## Introduction

Ruminants are a major source of meat production in China and are important for food security. Xinjiang Uygur Autonomous Region (XUAR) is located in northwest China and is a major ruminant production province. In 2017, beef production (0.43 million tons) and mutton production (0.58 million tons) in Xinjiang, respectively, accounted for the 6.8% and 12.4% in the total beef and mutton production in China. Ili is located in the western part of XUAR, where the economy is highly dependent on animal production [1, 2]. The combined number of cattle, sheep and goats is approximately 5.88 million in this region, in which the goats only accounted for the 2.1% because of economic value. The sheep, goats and cattle are reared under traditional systems, and confined sheep/goats or cattle ranches are the two main feeding systems.

Diseases and poor animal health are major risk factors for animal production in Ili. The viability of sheep and cattle production is largely determined by their reproductive ability, which is influenced by both genetic and environmental factors [3, 4]. Abortion is a serious threat to livestock, and it is also a public health issue as it is often induced by zoonotic microorganisms [5, 6].

Most ruminants are maintained by poor farmers as a way to increase family income. Abortion in sheep and dairy cows has a great impact on the animal production and the health of rural economies [7, 8]. The farming system and communal grazing are often involved in the spread of infectious organisms. There is a need for an improved diagnostics and specific control strategies for maintaining healthy livestock and public health safety [6]. Risk factors responsible for livestock abortion can be classified into infectious and non-infectious [9].

Infectious agents are the main causes of abortion in sheep and cattle as compared to non-infectious agents and are generally infectious to humans. The main etiological agents causing sheep and cattle abortion are *Brucella*, *Salmonella*, *Mycoplasma*, *Chlamydia abortus* and *Toxoplasma gondii* [9–12].

Ili is an endemic area for brucellosis with high incidences in sheep (4.21%) and cattle (6.91%) brucellosis in 2015 (Data from the Center for Animal Disease Control and Prevention of Ili). In this region, most farmers practice mixed farming (both sheep and dairy cows) and use a communal grazing system. Grazing in this environment can expose pregnant animals to pathogens [5, 13].

In recent years, the number of livestock abortions increased, according to the local Veterinary Department report. However, the causes of these abortions remained unknown. Hence, to protect and sustain the ruminant industry in the Ili region, we need to understand all of the reasons for animal abortion. Thus, the objectives of this study were to: 1) investigate the prevalence of abortion in Ili ruminant flocks and correlated its association with infectious agents (*Brucella*, *Salmonella*, *Mycoplasma* and *Chlamydia abortus*) and management (farming type and contact with other flocks) risk factors; 2) isolate and analyze genetic characteristics of the abortion-related pathogens.

## Materials and methods

### Ethics statement

All animals used in our experiment were treated humanely and in accordance with institutional animal care guidelines. Our study was approved by the Animal Care and Use Committee of Shihezi University.

### Study design

A cross-sectional study was carried out in three counties of the Ili region (X county, H county and Q county) between March and July in both 2017 and 2018. Samples from cows and sheep were collected from smallholder farms. The selection strategies including regions, villages and farms were described in Arif et al [14]. These three counties were selected mostly based on operational convenience, but they also represent a range of agro-ecological zones. Villages within each county were selected randomly by an electronic calculation.

### Herd selection

A total of 325 farms were selected from 25 villages in the three counties, and, given their availability, a maximum of five cows or sheep were randomly sampled in each farm. All of the livestock owners involved were informed about the purpose of this study and provided information about previous vaccinations. The study sampled non-vaccinated animals over two years of age. When there were more than five animals of the required age, five animals were selected randomly from the animals available.

### Sample size

The study population included all the farms in selected villages, but the target population was all of the cows and sheep within the selected villages and all of the villages in selected counties. Several studies reported that *Brucella* is a main pathogen responsible for animal abortion in XUAR [15–17]. Therefore, the sample size was calculated according to the estimated prevalence of brucellosis in these three counties, and the assumed prevalence is listed in Table 1. The minimum sample number of sheep and cows required assumed a closed population, as described previously [18]. The sample size of cows and sheep was estimated to detect a reduction of at least 4% for cows and 6% for sheep brucellosis prevalence with a confidence of 95% and a power of 80% according the following equation [18]:

$$n=\frac{{\left\lfloor Z\alpha \sqrt{2pq}-Z\beta \sqrt{p1q1+p2q2}\right\rfloor }^{2}}{{\left(p1-p2\right)}^{2}}$$

**Table 1:** The estimation of minimum required number in this study.

County	Expect prevalence		Desire prevalence		Minimum require number	
	Cow	Sheep	Cow	Sheep	Cow	Sheep
X	9.5%	8.3%	2%	3%	150	296
H	8.1%	6.7%	2%	3%	201	316
Q	5.5%	8.8%	1.5%	3%	330	261

In this equation, *n* is the minimum number of samples required, Z**α** represents the value obtained from standard normal distribution for 95% confidence (1.96), Z**β** represents the value obtained from standard normal distribution for a power of 80% (-0.84), *p*1 represents the estimated prevalence, an expected prevalence for cow and sheep brucellosis in these three counties (listed in Table 1), *p*2 represents the desired brucellosis prevalence for cows and sheep (listed in Table 1), *q*1 is (1-*p*1), *q*2 is (1-*p*2), *p* is (*p*1+*p*2)/2, and *q* is 1-*p*. The minimum number of cows and sheep required in these three counties is shown in Table 1.

### Sample collection

A total of 2996 blood samples (1406 dairy cows and 1590 sheep) were collected from jugular veins using venoject needles (Venoject, China) and stored in 5 mL sterile vacutainer tubes. Additionally, 141 aborted fetuses (66 cow fetuses and 75 sheep fetuses) and 65 milk samples (42 cow milk and 23 ewe milk) were collected. Blood samples were centrifuged at 3000 rpm for 10 min, and the serum was separated into a new sterile tube and stored at -20°C until tested. The milk samples were transported to the laboratory and stored in 4°C. The aborted fetuses were stored at -20°C until processing.

### Laboratory testing

All of the serum samples were screened for antibodies by RBPT and c-ELISA. Briefly, 30 μL of antigen was mixed with 30 μL of serum on a clean plate. After 3 min, any visible agglutination was considered as positive, and no agglutination was considered as negative. Positive or doubtful samples identified by RBPT were further tested with c-ELISA using the Svanovir *Brucella*-Ab-c-ELISA kits (Svanova Biotech, Uppsala, Sweden) according to the manufacturer’s instructions. The optical density (OD) of each samples were tested twice to obtain the average OD. The cutoff OD of 0.3 was used to identify positive reactions [19]. The sensitivity and specificity of these two methods have been validated as useful tools for brucellosis screening [20, 21]. Additionally, all of the serum sample were screened using the ELISA method to evaluate the changes of *Chlamydia abortus*-specific antibody titer. The mean value of OD was used to identify infected or non-infected livestock [22] and the *Mycoplasma bovis*-specific antibody concentration was determined by *Mycoplasma bovis* MilA IgG ELISA as described previously [23]. The antibodies against *Salmonella spp* were identified using an indirect ELISA kit as described previously [24].

### Risk factors questionnaire

A questionnaire was filled out by participating farm owners. The questionnaire contained information about abortion history in the livestock during the previous two years, livestock management risk factors including history of contact with other animals (yes or no) and type of farming; sheep flocks (containing only sheep), cow herds (containing only cows) or mixed groups (containing both sheep and cows).

### PCR examination

Samples of spleen, liver and stomach contents were collected aseptically from aborted fetuses of sheep or cows. The DNA extractions from tissue samples were performed using the TIANamp Genomic DNA Kit (TIANGEN BIOTECH CO., LTD, China) according to the manufacturer’s instructions. Nucleic acid extraction from raw milk was performed as previously described [25]. All of the samples were examined by PCR and the PCR primers used in this study are listed in S1 Table of the Supplementary Material.

### Pathogen isolation

The *Brucella* was isolated from raw milk as previously described [26, 27]. Spleen, liver and stomach contents were crushed and cultured on *Brucella* serum dextrose agar composed of *Brucella* medium base (supplemented with *Brucella* selective antibiotic, OXOID, England) and 5%-10% heat-inactivated horse serum (GIBCO, New Zealand). Plates were incubated with, and without, 5%-10% carbon dioxide at 37°C after inoculation with sample materials. The plates were examined after 3–5 d for bacterial growth. A single clone was chosen for identification. The *Salmonella spp*., *Mycoplasma bovis* and *Chlamydia abortus* were isolated from aborted fetuses or milk samples as described previously [28–30].

### Identification of isolates

The obtained single bacterial clones were identified using PCR targeting the 16S *rRNA* gene [31]. The PCR primers for examination of *Salmonella spp*., *Mycoplasma bovis* and *Chlamydia abortus* are listed in S1 Table of the Supplementary Material. The IS*711* PCR primers were used to identify the species of *Brucella*. PCR products purification and sequencing was conducted as described above. Phylogenetic analysis of isolates was done according to the IS*711* sequence. The sequence distance was determined by the neighbor-joining (NJ) method, and maximum-likelihood algorithms were analyzed using the Molecular Evolutionary Genetics Analysis (MEGA) 7 software [32]. The *Brucella* isolates were characterized by biochemical testing according to the standard strain identification method [33]. The carbon dioxide (CO_2_) requirement was tested on *Brucella* serum dextrose agar with and without CO_2_ during the first isolation. Agglutination by A, M and R monospecific antisera was detected by mixing the antisera with the isolate after dilution of the colony. This process was completed at the Center for Disease Prevention and control (CDC) of China in Beijing.

### Estimation of true prevalence

An animal was considered seropositive if it was positive on both the RBPT and c-ELISA, and a herd was considered positive if it contained at least one seropositive animal. Data were saved in Microsoft Excel and used for risk factors analysis and prevalence calculations.

The true prevalence for the study area was estimated using the software *Epitools* according to the method described by Rogan and Gladen [34]. True prevalence was estimated using the common sensitivity of RBPT and c-ELISA tests [35] and the specificity of c-ELISA (0.996) test. The common sensitivity was estimated as 0.981, which was the outcome of RBPT (0.986) [36] and c-ELISA (0.995) (Svanova Biotech, Uppsala, Sweden).

### Statistical analysis

To analyze the risk factors, a preliminary analysis of the data (univariate) was conducted to select the variables with *P* ≤ 0.05 by Chi-square test or Fisher’s exact test. Subsequently, the *P* ≤ 0.05 of variables was analyzed by multivariable logistic regression [37]. The collinearity was verified between each of the independent variables by correlation analysis, and a correlation coefficient >0.9 indicated the variables with strong collinearity. Because of the problem of multicollinearity, one or two variables were excluded from the multiple analysis based on the biological plausibility [38]. Confounding data were evaluated by adding new variables and then monitoring the changes in the model parameters. Large changes (>20%) in the regression coefficients were considered indicative of confounding. The calculations were made using SPSS software 17.0.

## Results

### Distribution of seroprevalence of four abortion-related pathogens in three counties

A total of 2,996 serum samples (1406 dairy cows and 1590 sheep) were collected from X county (cow 354, sheep 351), H county (cow 342, sheep 361) and Q county (cow 710, sheep 878) and then identified by RBPT, c-ELISA and ELISA. The overall brucellosis positivity for cows and sheep in the study area was cow 6.76%, sheep 9.50%, which was the highest rate among the four abortion-related diseases in this study (Table 2). The brucellosis positivity for cows and sheep in X county was cow 7.06%, sheep 9.12%; in H county was cow 11.70%, sheep 10.80%; and in Q county was cow 4.22%, sheep 9.11%, which is much higher than the seroprevalence of other pathogens in these three counties (Table 2). However, our results suggest that the *Mycoplasma* infection is an additional threat to livestock reproduction. The overall *Mycoplasma* positivity for cows and sheep in the study area was cow 3.20%, sheep 6.42% (Table 2). The *Mycoplasma* positivity for cows and sheep in X county was cow 3.39%, sheep 7.98%; in H county was cow 5.26%, sheep 9.97%; and in Q county was cow 2.11%, sheep 4.33% (Table 2), and its abortion rate for cows and sheep was 26.60% (12/45, *P* = 0.003) and 30.40% (31/102, *P*<0.001) (Table 3). The salmonellosis and *Chlamydia abortus* seroprevalence for cows and sheep in these three counties are shown in Table 2.

**Table 2:** Seroprevalence of brucellosis, salmonellosis, *Mycoplasma* and *Chlamydia abortus* in three counties.

Variables	X County	H County	Q County	Overall
Brucellosis positivity				
Cow	25/354 (7.06%)	40/342 (11.70%)	30/710 (4.22%)	95/1406 (6.76%)
Sheep	32/351 (9.12%)	39/361 (10.80%)	80/878 (9.11%)	151/1590 (9.50%)
Salmonellosis positivity				
Cow	3/354 (0.85%)	4/342 (1.17%)	3/710 (0.42%)	10/1406 (0.71%)
Sheep	6/351 (1.71%)	6/361 (1.66%)	4/878 (0.46%)	16/1590 (1.01%)
*Mycoplasma* positivity				
Cow	12/354 (3.39%)	18/342 (5.26%)	15/710 (2.11%)	45/1406 (3.20%)
Sheep	28/351 (7.98%)	36/361 (9.97%)	38/878 (4.33%)	102/1590 (6.42%)
*Chlamydia abortus* positivity				
Cow	1/354 (0.28%)	1/342 (0.29%)	3/710 (0.42%)	5/1406 (0.36%)
Sheep	2/351 (0.57%)	1/361 (0.28%)	4/878 (0.46%)	7/1590 (0.44%)

**Table 3:** Univariable analysis of abortion-related factors of livestock in the Ili region.

Variables	No. of livestock sampled	No. of livestock with abortion	Rate of abortion	*P*-*value*
County				
X	705	106	15.03%	0.245
H	703	86	12.20%	
Q	1588	204	12.80%	
Type of farming				
Cow	160	8	5.00%	0.016*
Mixed (Cow)	203	25	12.30%	
Sheep	141	11	7.80%	0.005*
Mixed (Sheep)	272	49	18.00%	
Contact with other flock				
No (Cow)	76	6	7.90%	0.008*
Yes (Cow)	284	60	21.10%	
No (Sheep)	114	11	9.60%	0.002*
Yes (Sheep)	331	75	22.70%	
Brucellosis positivity				
No (Cow)	1311	99	7.55%	<0.0001*
Yes (Cow)	95	75	78.90%	
No (Sheep)	1439	96	6.67%	<0.0001*
Yes (Sheep)	151	126	83.44%	
Salmonellosis positivity				
No (Cow)	1396	174	12.46%	0.233
Yes (Cow)	10	0	0%	
No (Sheep)	1574	222	14.10%	0.105
Yes (Sheep)	16	0	0%	
*Mycoplasma* positivity				
No (Cow)	1361	162	11.90%	0.003*
Yes (Cow)	45	12	26.60%	
No (Sheep)	1488	191	12.83%	<0.001*
Yes (Sheep)	102	31	30.40%	
*Chlamydia abortus* positivity				
No (Cow)	1401	174	12.41%	0.400
Yes (Cow)	5	0	0%	
No (Sheep)	1583	222	14.02%	0.285
Yes (Sheep)	7	0	0%	

* Variables selected and subjected to the multiple analysis (*P*<0.05)

### Other livestock management factors involved in abortion

Univariable analysis of abortion-related risk factors (Table 3) found no significant differences among the studied counties (*P* = 0.245); the abortion rate in the three regions ranged from 12.20% to 15.03%. However, the management factors were significantly correlated with sheep or cow abortion including the type of farming (cow *P* = 0.016, sheep *P* = 0.005) and contact with other herds or flocks (cow *P* = 0.008, sheep *P* = 0.002). Among the four pathogens, *Brucella* was the main reason for cow or sheep abortion, and the abortion rate of cow or sheep brucellosis was 78.90% and 83.44% (*P*<0.0001) (Table 3). *Mycoplasma* infection also posed a threat to cow and sheep reproduction, and the abortion rates were, respectively 26.60% (*P* = 0.003) and 30.40% (*P*<0.001) (Table 3).

### Brucellosis is the main factor responsible for cow and sheep abortion

The abortion-related risk factors analyzed through multivariable logistic regression showed that brucellosis was the biggest risk factor for livestock abortion (Table 4). Our results also showed the brucellosis positivity was significantly associated with cow (*P*<0.001) and sheep (*P*<0.001) abortion in the Ili region, and its abortion rates for cow and sheep, respectively, were 78.9% (75/95) and 83.44% (126/151) (Table 3). The Exp (B) values of brucellosis for cow and sheep, respectively, were 45.909 [95% CI 26.912–78.317, *P*<0.001] and 70.507 [95% CI 43.783–113.544, *P*<0.001] times higher than other abortion-related factors including mixed farming, contact with other flocks and *Mycoplasma* infection (Table 4).

**Table 4:** Abortion-related risk factors of livestock in the Ili region.

Risk factors	Logistic regression coefficient	Standard error	Wald	Exp(B)	95% CI	*P*-value
Mixed farming						
Cow	0.982	0.421	5.437	2.669	1.169–6.090	0.02
Sheep	0.954	0.351	7.374	2.597	1.304–5.171	0.007
Contact with other flock						
Cow	1.139	0.450	6.425	3.125	1.295–7.542	0.011
Sheep	1.009	0.343	8.641	2.743	1.400–5.376	0.003
Brucellosis positivity						
Cow	3.827	0.273	197.192	45.909	26.912–78.317	<0.001
Sheep	4.256	0.243	306.461	70.507	43.783–113.544	<0.001
*Mycoplasma* positivity						
Cow	0.990	0.347	8.125	2.691	1.362–5.316	0.004
Sheep	1.087	0.229	22.564	2.965	1.893–4.643	<0.001

Exp(B) represent the Odds ratio.

### Molecular detection

In the present study, all of the 75 aborted sheep fetuses, 66 aborted cow fetuses, 42 milk and 23 ewe milk samples were screened by PCR targeting the 16s *rRNA* gene. A total of 54 samples (22 aborted cow fetuses, 30 aborted sheep fetuses, 1 milk and 1 ewe’s milk) were positive and were further identified as *B*. *melitensis* by targeting the IS*711* gene (data not shown). However, all of these samples were negative for *Salmonella spp*., *Mycoplasma bovis* and *Chlamydia abortus* identified by PCR. The nucleotide sequences from this study have been deposited in the GeneBank database (IS*711*: MK913893-MK913898).

### Identification of isolates

A total of 38 (70.37%) *Brucella* isolates were isolated from 54 positive samples, including 20 aborted sheep fetuses, 16 aborted cow fetuses and 1 milk sample and 1 ewe’s milk sample (Table 5). All of the isolates were positive for 16S *rRNA*. The *Brucella* differentiation was performed by PCR utilizing primers specific to the IS*711* gene of *B*. *melitensis*. *B*. *melitensis*-specific DNA fragments with 731 bp were amplified from all isolates, and no DNA was observed in negative control samples. Only part of the results is presented in S1 Fig. Furthermore, all of the isolates were identified as *B*. *melitensis* biovar 3 by biochemical testing. The growth of all the 6 isolates on a medium with thionin at 40 μg/mL (1:25000) concentration and basic fuchsin at all concentrations suggested that these isolates were *B*. *melitensis* biovar 3. Only part of the results is presented in Table 6. No *Salmonella spp*., *Mycoplasma bovis* and *Chlamydia abortus* were isolated from aborted fetuses and milk samples.

**Table 5:** Comparison of PCR and culture results from aborted cow, sheep fetuses and milk samples.

No. of samples	Host	PCR results	Culture results
1–16 fetuses	cow	+	+
17–22 fetuses	cow	+	-
1 milk	cow	+	+
1–20 fetuses	sheep	+	+
21–30 fetuses	sheep	+	-
1 milk	sheep	+	+

**Table 6:** Species and biovar differentiation of the *Brucella* isolates.

*Brucella* isolates	Source	Growth characteristics					Monospecific sera		Phage typing						Interpretation
		Urea	H_2_S	CO_2_	BF	TH	A	M	Tb	Wb	BK_2_	Fi	Iz	R/C	*B*. *melitensis biovar 3*
DXY1	Fetal spleen	++	-	-	+	+	+	+	NL	NL	CL	NL	PL	NL	*B*. *melitensis biovar 3*
DXY3	Fetal liver	++	-	-	+	+	+	+	NL	NL	CL	NL	PL	NL	*B*. *melitensis biovar 3*
DXY6	Milk	++	-	-	+	+	+	+	NL	NL	CL	NL	PL	NL	*B*. *melitensis biovar 3*
DXY8	Ewe’s milk	++	-	-	+	+	+	+	NL	NL	CL	NL	PL	NL	*B*. *melitensis biovar 3*
DXY5794	Stomach content	++	-	-	+	+	+	+	NL	NL	CL	NL	PL	NL	*B*. *melitensis biovar 3*
DXY1954	Stomach content	++	-	-	+	+	+	+	NL	NL	CL	NL	PL	NL	*B*. *melitensis biovar 3*

BF: basic fuchsin at 20 μL/mL (1/50,000 w/v), TH: thionin at 20 μL/mL (1/50,000 w/v), CL: confluent lysis, PL: partial lysis, NL: no lysis.

### Phylogenetic analysis

**Fig 1:**
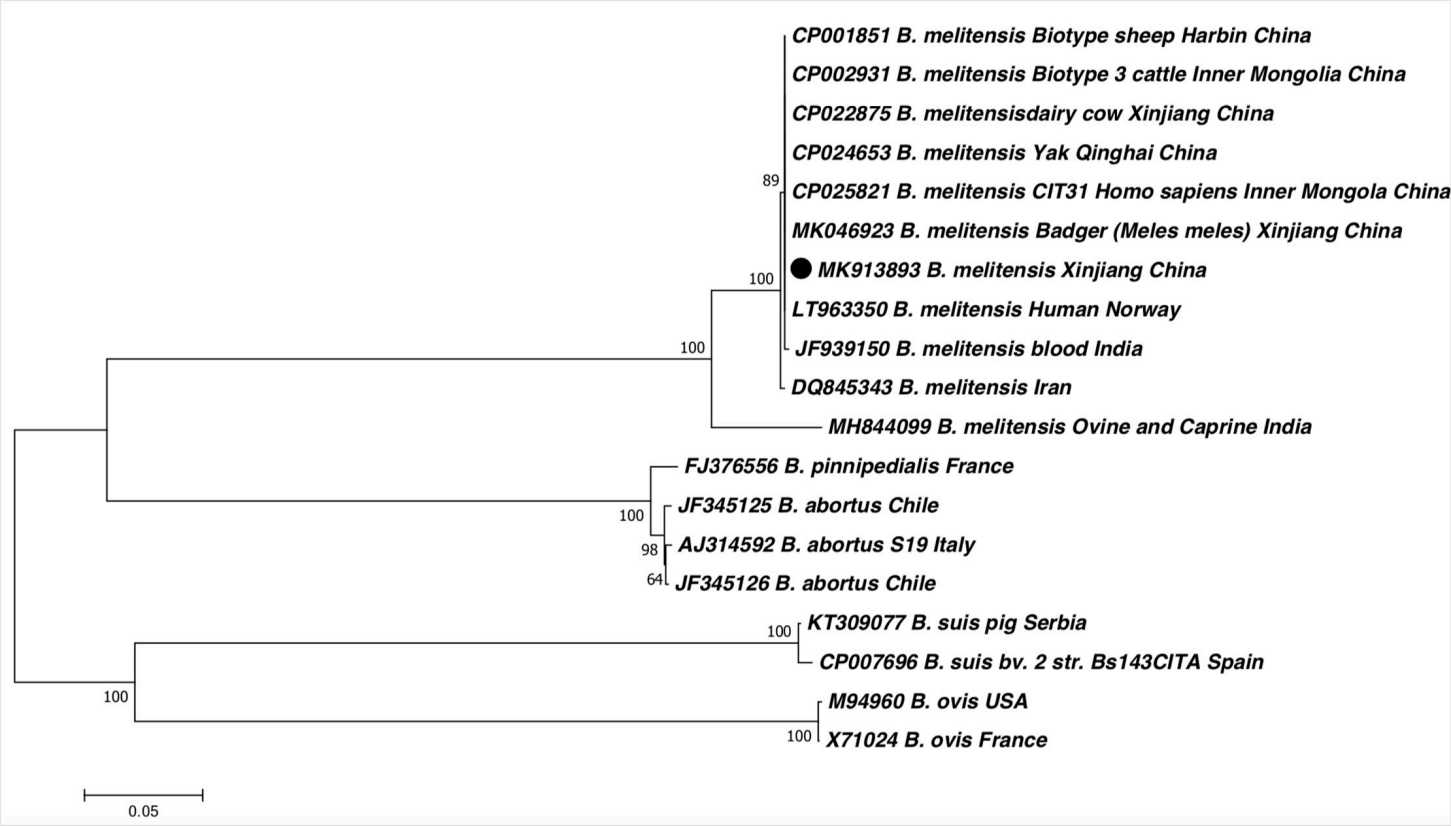
Phylogenetic tree of the IS*711* concatenated sequence of *Brucella melitensis* (⦁) isolated from aborted cow or sheep fetuses in this study and reference sequences from *Brucella melitensis* retrieved from the GenBank database. The tree was constructed according to the neighbor-joining (NJ; 500 bootstrap replicates) and maximum–likelihood (ML, 1000 bootstrap replicates) analyses using MEGA7. The scale bar represents the inferred substitutions per nucleotide site.

A phylogenetic tree was constructed based on the 731 bp sequence of the IS*711* repetitive element for all isolates. After sequencing, we found that the IS*711* gene sequences from all of these isolates showed 100% similarity (731/731bp). Phylogenetic analysis showed that the *Brucella* isolates closely matched those of *B*. *melitensis* biovar 3 isolated from cattle in Inner Mongolia, China. Isolates from Norway and India also showed 100% similarity to the isolates of the present study in clade 1 (Fig 1). The isolates of *B*. *melitensis* from other countries were placed into different clades based on low similarity to the *Brucella* isolates from this study (Fig 1).

## Discussion

The livestock industry of the XUAR is a major source of its economic growth especially in some remote areas like Ili. However, there are few studies on the prevalence of brucellosis in this region. It has been reported that the brucellosis seroprevalence for cattle and sheep in Ili region was cattle 1.72%, sheep 1.95% in 2014 [39]. According to the data released from the Center for Animal Disease Control and Prevention of Ili in 2015, the brucellosis seroprevalence for cows and sheep were 6.91% and 4.21%. Although, the local control strategy was to vaccinate livestock for preventing brucellosis, animal brucellosis still occurred in an increasing number of cases in recent years. In the present study, we investigated the seroprevalence of abortion-related pathogens (*Brucella*, *Salmonella*, *Mycoplasma* and *Chlamydia abortus*) in three counties (X, H and Q). A total of 2996 cow and sheep serum samples were screened by RBPT, c-ELISA and ELISA. The resulting data showed that the brucellosis was widely prevalent in livestock in all of the studied counties. The seroprevalence for cows and sheep in X county was cow 7.06%, sheep 9.12%; in H county was cow 11.70%, sheep 10.80%; and in Q county was cow 4.22%, sheep 9.11% (Table 2). These data suggest that the disease is distributed within all of the Ili region and can potentially infect all of the susceptible livestock in this region. The results showed that *Mycoplasma* infection was also present and this can influence the livestock reproduction, although its seroprevalence was not as high as brucellosis. The seroprevalence for *Mycoplasma* infection in X county was cow 3.39%, sheep 7.98%; in H county was cow 5.26%, sheep 9.97%; and in Q county was cow 2.11%, sheep 4.33% (Table 2). The abortion rates of *Mycoplasma* positivity for cow and sheep were, respectively, 26.60% (12/45) and 30.40% (31/102) (Table 3). However, Wenhao Ni et al. [40], found that in 2018, the seroprevalence rates of *Mycoplasma* for Hazake sheep and Suffolk sheep were 22.2% and 8.3% in the Ili region. These data are similar to our results except that the higher seroprevalence in Hazake sheep may a breed-related difference.

Many reasons could induce abortion in pregnant animals include infectious factors and non-infectious factors, in which infectious factors include *Brucella*, *Salmonella spp*., *Mycoplasma bovis*, *Chlamydia abortus*, *Listeria monocytogenes*, bovine viral diarrhea virus (BVDV), *Neospora enterica* and *T*. *gondii* [10–12, 41, 42] and non-infectious factors involve heat stress, production stress, seasonal effect, chromosomal and single gene disorders [43–46]. We previously have found that the *B*. *melitensis* biovar 3 was the main cause of cow and sheep abortion in Nilka county (neighboring X county) in 2016 [47]. However, we could not rule out aborted fetuses caused by non-infectious factors, viral and parasitic agents, because we only examined bacterial agents in this study. In addition to the effects of pathogens on livestock abortion, we found that livestock abortion can also be influenced by livestock management systems including herd and flock size, mixed farming, grazing system and contact with other animals [48, 49]. We used univariable analysis to study management risk factors related to livestock abortion in the Ili region and found statistically significant links with the type of farming (cow *P* = 0.016, sheep *P* = 0.005) and contact with other herds/flocks (cow *P* = 0.008, sheep *P* = 0.002) (Table 3). This may be because these two management factors are easily overlooked by livestock owners.

Bacterial isolation is the gold standard for the diagnosis of brucellosis. We isolated a total of 38 *B*. *melitensis* biovar 3 isolates from 16 aborted cow fetuses, 20 aborted sheep fetuses and 1 milk and 1 ewe’s milk sample (Table 5). However, the 16 aborted fetuses that were positive for PCR but negative for culture probably occurred because contamination decreased the rate of *Brucella* isolation. Interestingly, all of the isolates from 16 aborted cow fetuses were identified as *B*. *melitensis* and 10 out of these 16 aborted cow fetuses were collected from mixed farming group. This finding is in agreement with previous report, 34 *B*. *melitensis* were isolated from cow aborted fetuses and milk in a farm in Ili region [47]. We also identified the *Salmonella spp*., *Mycoplasma* and *Chlamydia abortus* through PCR. But, no aborted fetuses were positive for these pathogens. These results show that *B*. *melitensis* biovar 3 is the dominant pathogen responsible for sheep and cow abortion.

RBPT and c-ELSA were combined to screen and diagnose brucellosis in China especially in some remote areas. The sensitivity and specificity of these two methods has been described previously [21, 50]. However, these two methods are not good tools for diagnosing brucellosis in the laboratory. We consider that the best way is bacterial isolation and identification. Molecular approaches appeared to be faster and more sensitive than traditional bacteriological tests [51, 52]. The 16S *rRNA* component of the 30S small subunit of prokaryotic ribosomes contains hyper-variable regions that provide species-specific signature sequences useful for bacterial identification. Therefore, the 16S *rRNA* gene can be used as the diagnostic target in the PCR for confirmatory identification of *B*. *melitensis* [53]. Several studies have demonstrated that the 16S *rRNA* can be used as a rapid tool for *Brucella* identification [53, 54]. In this study, we identified 38 *Brucella* isolates with PCR by targeting the 16S *rRNA* gene in the first round of screening and these were further identified as *B*. *melitensis* by the presence of the IS*711* gene. The advantage of this method is that results can be obtained within one day as compared to seven days using traditional microbiological testing.

Brucellosis is principally an animal disease, but >500,000 human cases are reported each year globally [55]. Transmission to humans occurs primarily through contact with infected animals and consumption of contaminated food such as raw milk and its byproducts [56]. This study discovered *B*. *melitensis* biovar 3 isolates in raw milk and ewe’s milk. This result suggests that *B*. *melitensis* infection in cows and ewes is a public health issue in China. Infected cows and ewes, as disease reservoirs, can spread contaminated milk to the local human population. We recommend: i) increasing the regular quarantine of brucellosis and timely elimination of infected ewes or cows and their products and ii) implementing a vaccination program for livestock and iii) reducing mixed farming and avoiding contact with other herds/flocks and encouraging livestock owners to learn and adopt new management skills.

## Conclusions

*B*. *melitensis* biovar 3 was identified as the main pathogen responsible for cow and sheep abortion. *Mycoplasma* infection, mixed farming and contact with other herds and flocks are strongly correlated with livestock abortion. An effective vaccination and control program is advocated for livestock owners in the Ili region to prevent the spread of brucellosis and *Mycoplasma* infection.

## Supporting information

S1 Table Primers used in this study. (XLSX)

S2 Table (XLSX)

S1 Fig PCR product of IS*711* gene amplication. (TIF)

S1 Questionnaire (XLSX)

S2 Questionnaire (XLSX)
