# Supplementary figures and images for: Abortion and various associated risk factors in dairy cow and sheep in Ili, China
Source: PLoS One. 2020 Oct 30;15(10):e0232568. doi: 10.1371/journal.pone.0232568 (PMC7598486; doi:10.1371/journal.pone.0232568)

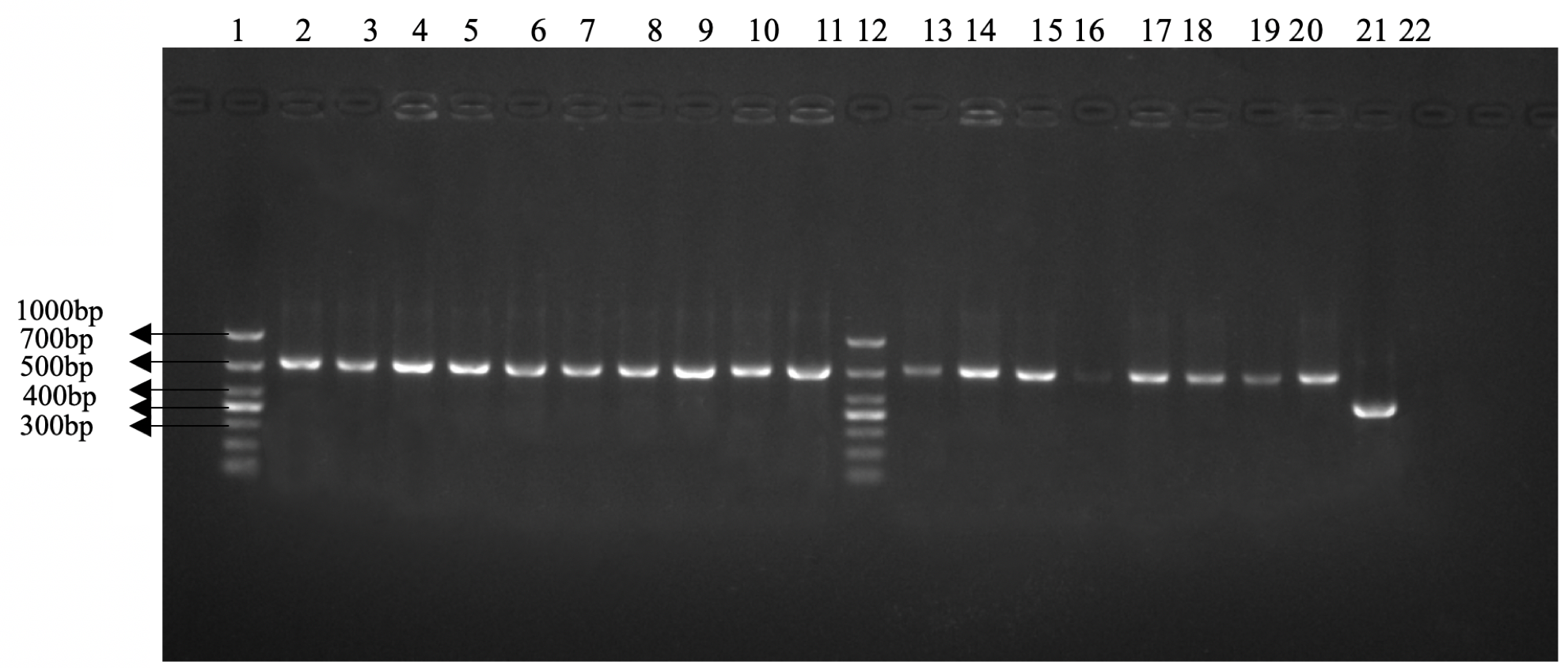

Supplement: S1 Fig — (TIF) [file pone.0232568.s003.tif]
